# High risk of HIV in non-brothel based female sex workers in India

**DOI:** 10.1186/1471-2458-5-87

**Published:** 2005-08-20

**Authors:** Rakhi Dandona, Lalit Dandona, Juan Pablo Gutierrez, Anil G Kumar, Sam McPherson, Fiona Samuels, Stefano M Bertozzi, the ASCI FPP Study Team

**Affiliations:** 1 Health Studies Area, Centre for Human Development, Administrative Staff College of India, Hyderabad, India; 2 Division of Health Economics and Policy, National Institute of Public Health, Cuernavaca, Mexico; 3 Research and Evaluation Unit, International HIV/AIDS Alliance, Brighton, UK; 4 CIDE, Mexico City, Mexico

## Abstract

**Background:**

Heterosexual contact is the most common mode of HIV transmission in India that is largely linked to sex work. We assessed the non-use of condoms in sex work and with regular sex partners by female sex workers (FSWs), and identified its associations that could assist in planning HIV prevention programmes.

**Methods:**

Detailed documentation of various aspects of sex work, and sexual behaviour with regular sex partners, was done through confidential interviews for 6648 FSWs in 13 districts in the Indian state of Andhra Pradesh. Multivariate analysis was done to understand condom non-use with clients.

**Results:**

5010 (75.4%), 1499 (22.5%), and 139 (2.1%) FSWs were street-, home-, and brothel-based, respectively. Of the total 6648 FSWs, 6165 (92.7%) had penetrative vaginal/anal sex with at least one client in the last 15 days, and of these 2907 (47.2%; 95% CI 41.2–53.2%) reported non-use of condom with at least one of her last three clients. Lack of knowledge that HIV could be prevented (odds ratio 5.01; 95% CI 4.38–5.73), no access to free condoms (odds ratio 3.45; 95% CI 2.99–3.98), being street-based as compared with brothel-based (odds ratio 3.36; 95% CI 1.87–6.04), and no participation in FSW support groups (odds ratio 2.02; 95% CI 1.50–2.70) were the most significant predictors of condom non-use with clients. Other associations included lower social support, lower income, age >24 years, illiteracy, and living in medium-size urban or rural areas. Of the 2582 who had penetrative sex with regular sex partner within the last 7 days, 2428 (94%; 95% CI 92.1–95.9%) had not used condom at last sex, and 1032 (41.8%) had neither used condom consistently with clients nor with regular sex partner.

**Conclusion:**

About half the FSWs do not use condom consistently with their clients in this Indian state putting them at high risk of HIV infection. Non-brothel-based FSWs, who form the majority of sex workers in India, were at a significantly higher risk of HIV infection as compared with brothel-based FSWs. With their high vulnerability, the success of expansion of HIV prevention efforts will depend on achieving and sustaining an environment that enables HIV prevention with the non-brothel based FSWs.

## Background

Around the world, the number of people living with HIV continues to rise despite the fact that effective prevention strategies exist. India has the largest number of people living with HIV, an estimated 5.1 million in the year 2003, after South Africa [1,2]. Heterosexual contact has been estimated to be the most common mode of transmission of infection in India, and six Indian states have been categorised as high prevalence states because HIV prevalence in these states exceeds 5% among the high-risk individuals and 1% among the women attending antenatal clinics [1]. In these six states, HIV is estimated to be transmitted through heterosexual sex to a large degree and is linked to sex work in four states of Andhra Pradesh, Karnataka, Maharashtra, and Tamil Nadu, and through injecting drug use in the other two states of Manipur and Nagaland [1,2].

Epidemiologically, great majority of new HIV infections in Asia occur in individuals who are at high risk – sex workers and their clients, men who have sex with men, and injecting drug users, and their immediate long-term sex partners [3-5]. Increasing prevalence of HIV in sex workers is an indication of increasing probability of a generalised epidemic [2]. A high prevalence of HIV in female sex workers (FSWs) has been reported recently from some parts of India, including the state of Andhra Pradesh [6,7] This occurrence means that adequately resourced efforts focused on achieving good coverage among those individuals at high risk of acquiring or transmitting HIV may prevent further spread of HIV in broader population. There is evidence that the HIV prevention programmes for FSWs can be highly effective in preventing HIV transmission [8-11]. Recent modelling to assess the impact of four types of interventions on prevention of HIV transmission in India has suggested FSW interventions that promote use of condoms in addition to other safe sex practices to be the most effective in preventing HIV transmission as compared with interventions focussing on treatment of sexually transmitted infections, prevention of mother-to-child transmission and provision of the highly active antiretroviral therapy [12].

Of the 835 government-supported targeted intervention programmes for high-risk individuals in India, 199 (23.8%) target FSWs and 171 (20.5%) target truckers, and the remaining target migrant workers, street children, prisons, men who have sex with men, intravenous drug users, and others [13]. These interventions follow a comprehensive approach to reduce HIV transmission and include behaviour change communication, counseling and provision of health care support, treatment for sexually-transmitted infections, and creation of an enabling environment to facilitate behaviour change [13]. One of the main foci of behaviour change in the HIV prevention efforts for FSWs is encouraging correct and consistent use of condom between them and their clients [2,8-11,13-16], as condoms reduce the risk of HIV transmission [17,18].

In this background, we assessed the non-use of condom for penetrative sex for a large sample of FSWs in the state of Andhra Pradesh, which is one of the high HIV prevalence states in India. This study was carried out as part of an impact assessment study of the Frontiers Prevention Project (FPP). The FPP aims to reduce the spread of HIV within the population through provision of HIV interventions in a geographically defined area that reduce risk behavior and STI prevalence among FSWs, men who have sex with men, and people living with HIV/AIDS by working in close collaboration with these population groups. FPP is being implemented in India (the state of Andhra Pradesh), Ecuador and Cambodia. We report data on condom use in sex work for penetrative sex between FSWs and their clients, and between FSWs and their regular sex partners in Andhra Pradesh, India.

## Methods

The objectives of this baseline study were to document the socio-demographic and sex work characteristics of FSWs, analyse these data to identify issues that needed particular attention for prevention of HIV and other sexually transmitted infections, and compare these baseline data later with a follow-up study to assess the impact of the FPP. The methods relevant to this paper are mentioned below.

This study was approved by the Ethics Committees of the Administrative Staff College of India, Mexico's National Institute of Public Health, the International HIV/AIDS Alliance, and by the Indian Health Ministry's Screening Committee, Indian Council of Medical Research, New Delhi. Permissions were obtained from the Andhra Pradesh State AIDS Control Society, the agency coordinating HIV/AIDS control activities in the state, to carry out the study.

### Study area

Forty geographic sites in 13 districts of the Telangana and Rayalseema regions of Andhra Pradesh state were identified where access to FSWs was considered feasible through non-governmental organisations having links with them. Each geographic site consisted of one or more close-by locations (cities/towns/villages) where FSWs were accessible. The total number of locations included in the 40 geographic sites were 72, of which 25 were rural and 47 were urban of various sizes, according to the Census of India definitions. [19]. The total sample size required of FSWs at the 40 geographic sites was estimated as 6,500 to detect a significant change in the various aspects of high-risk sexual behaviour between the baseline and follow-up studies. The sample size of the FSWs in each site was aimed to be proportional to their estimated number and type in that site, which was based on enumeration with the help of FSWs.

### Data collection

Data collection questionnaire was developed by an international team with multidisciplinary background through review of worldwide literature including previously used questionnaires, focus group discussions and in-depth interviews with FSWs to better understand the local context in Andhra Pradesh, and pre-pilot studies were conducted to capture a variety of issues related to the socio-economic context of FSWs, the sexual practices between FSWs and their clients and with regular sex partners, and awareness about HIV and sexually transmitted infections. An international technical advisory group provided input regarding the refinement of questionnaire. The questionnaire was developed in English, was translated in Telugu, the local language, following which it was back-translated into English in order to ensure accurate and relevant meaning and intent of the questions. Extensive training of the interviewers was done by a variety of survey experts and FSW representatives in order to address technical and ethical issues as well as to promote cultural sensitivity.

Data were collected between July 2003 and April 2004. At each study location, FSW facilitators helped contact and recruit FSW respondents more than 15 years of age for this study. Standardised procedures were followed for contacting respondents, which included approaching them with the help of FSW facilitators (peers) and convincing them of the confidentiality of the interviews. Written informed consent for participation was obtained from each respondent. Trained interviewers did one-to-one interviews confidentially in private settings that were selected in consultation with FSW facilitators. The names of respondents were not recorded and hence cannot be linked to the data. The data collection process in the field involved supervision of the work of interviewers by a Quality Control Supervisor and a Field Manager in each of the two field teams. Data were entered in an LSD (Sistemas Integrales, Santiago, Chile) database, and all data entries made by a data entry operator were fully checked by another operator to minimise errors in data entry.

### Data analysis

SPSS software was used for data analyses, and the different types of FSWs were defined as:

• *Street-based FSWs *if they primarily solicited their clients on streets (such as cinema, park, bus-stand, railway station, hotel / lodge) and provided services at hotel/lodge or a place of client's choice.

• *Home-based FSWs *if they primarily solicited their clients at home either directly or through a mediator and provided services at their homes.

• *Brothel-based FSWs *if they primarily solicited clients through an agent (such as pimp, madam) or mediator and provided services at a brothel. Brothel was defined as a place of sex work with at least 2 FSWs working under control of an agent.

The main outcome variable assessed in this analysis was the no or inconsistent use of condom for penetrative vaginal/anal sex between FSWs and their clients. Non-use of condom with all the last 3 clients or at least with one of the last 3 clients was considered as no or inconsistent condom use for this analysis. The 95% confidence intervals of these estimates of condom non-use were adjusted for the design effect of cluster sampling, based on intra cluster variance for these variables [20]. Univariate and multivariate analyses were done to understand the association of no or inconsistent use of condom for penetrative vaginal/anal sex with clients with other characteristics to identify those that may play a significant role in determining the use of condom. In the multiple logistic regression model, the effect of each category of a multi-categorical variable was assessed by keeping the first or the last category as reference. All the variables were introduced simultaneously in the model. Possible interactions between different variables in the model were assessed, where necessary. Use of condom for the last penetrative vaginal/anal sex between FSWs and their regular sex partners was also assessed. Regular sex partner was defined as a man who was not a client and with whom the FSW had sexual contacts.

## Results

### FSW characteristics

A total of 7251 FSW were contacted of whom 6648 (91.7%) participated in the study. Among these, 5010 (75.4%), 1499 (22.5%), and 139 (2.1%) were street-, home-, and brothel-based FSWs, respectively. The age range of FSWs was 16 to 54 years with mean age of 27.3 years, 2698 (40.6%) were currently married, 2833 (42.6%) were previously married, 1117 (16.8%) were never married, 4966 (74.7%) had no schooling, and 3105 (46.7%) were also involved in work other than sex work.

#### Sex work

Details of sex work are summarised in Table 1 for the different types of FSWs. Among the 5851 (88.%) FSWs who had worked in the last 7 days from the day of interview, the number of paying clients ranged from 1 to 49 with the mean number of paying clients being 7.5 in those 7 days. The income from sex work in these 7 days ranged from Rs. 10 to 13000, and the mean income per day was Rs. 96 (US$ 2.1). Street-based FSWs had lower income from sex work as compared with the home- and brothel-based FSWs.

**Table 1:** Distribution of variables related to sex work for the different types of FSWs.

		Type of female sex workers*			
		
**Variable**	**Variable categories**	**Street-based (N = 5010) Number (% of N)**	**Home-based (N = 1499) Number (% of N)**	**Brothel-based (N = 139) Number (% of N)**	**Total (N = 6648) Number (% of N)**
**Age at starting sex work (years)**	12–15	191 (3.8%)	356 (23.7%)	33 (23.7%)	580 (8.7%)
	16–19	955 (19.1%)	394 (26.3%)	43 (30.9%)	1392 (20.9%)
	20–24	1643 (32.8%)	334 (22.3%)	40 (28.8%)	2017 (30.3%)
	25–29	1475 (29.4%)	273 (18.3%)	15 (10.8%)	1763 (26.5%)
	30–34	578 (11.5%)	109 (7.3%)	5 (3.6%)	692 (10.4%)
	> = 35	168 (3.4%)	32 (2.1%)	3 (2.2%)	203 (3.1%)

**Duration of being in sex work (years)**	1 year or less	916 (18.3%)	174 (11.6%)	23 (16.5%)	1113 (16.7%)
	1.1 – 2.0	1060 (21.2%)	195 (13.0%)	18 (12.9%)	1273 (19.1%)
	2.1 – 3.0	892 (17.8%)	234 (15.6%)	20 (14.4%)	1146 (17.2%)
	3.1 – 4.0	629 (12.6%)	186 (12.4%)	16 (11.5%)	831 (12.5%)
	4.1 – 5.0	567 (11.3%)	196 (13.1%)	23 (16.5%)	786 (11.8%)
	More than 5	946 (18.9%)	514 (34.3%)	39 (28.1%)	1499 (22.5%)

**Number of months practiced sex work in the last 12 months**	0–6	409 (8.2%)	59 (3.9%)	12 (8.7%)	480 (7.2%)
	7–9	583 (11.6%)	127 (8.5%)	8 (5.8%)	718 (10.8%)
	10–12	4016 (80.2%)	1312 (87.6%)	118 (85.5%)	5446 (82%)

**Number of paying clients in the last 7 days**	None	638 (12.7%)	151 (10.1%)	8 (5.8%)	797 (12.0%)
	1–2	631 (12.6%)	156 (10.4%)	5 (3.6%)	792 (11.9%)
	3–7	2159 (43.1%)	495 (33.0%)	28 (20.1%)	2682 (40.3%)
	8–14	1322 (26.4%)	471 (31.4%)	46 (33.1%)	1839 (27.7%)
	15–28	248 (5.0%)	220 (14.7%)	49 (35.3%)	517 (7.8%)
	More than 28	12 (0.2%)	6 (0.4%)	3 (2.2%)	21 (0.3%)

**Income in the last 7 days from sex work (in Indian Rupees)**	None	637 (12.7%)	151 (10.1%)	9 (6.5%)	797 (12.0%)
	250 or less (US $5.5 or less)	1688 (33.7%)	292 (19.5%)	14 (10.1%)	1994 (30.0%)
	251 – 500	1551 (31.0%)	360 (24.0%)	29 (20.9%)	1940 (29.2%)
	More than 500	1132 (22.6%)	696 (46.4%)	87 (62.6%)	1915 (28.8%)

**Participation in FSW support group**	Yes	433 (8.6%)	152 (10.1%)	15 (10.8%)	600 (9.0%)
	No	4576 (91.4%)	1347 (89.9%)	124 (89.2%)	6047 (91.0%)

**Family aware of sex work**	Yes	1157 (23.1%)	954 (63.6%)	69 (49.6%)	2180 (32.8%)
	No	3836 (76.6%)	542 (36.2%)	69 (49.6%)	4447 (66.9%)
	Refused to answer	14 (0.3%)	3 (0.2%)	1 (0.7%)	18 (0.3%)

*The total of sub-categories may not always be 5010, 1499, and 139 due to a few missing values for street-, home-, brothel-based FSWs, respectively.

Of the 6648 FSWs, 1594 (23.9%; 95% CI 19.4–28.6%; design effect 20.2) reported having never used condom. Among the 5010 FSWs who reported having ever used condom, 2942 (58.7%) reported having access to free condoms and 2468 (83.9%) of these had received condoms for free within the last 30 days from the day of interview. Non-governmental organisations were the main source of free condoms (71.6%) followed by clinic/hospital (10.3%). The other sources reported were condom outlet box, another FSW/peer educator, and pimp/madam.

### Client characteristics

Data were documented for each FSW on her last three clients (if she had that many clients) within the 15 days from the day of interview. Among the 6648 FSWs, 6,171 (92.8%) had at least one client in the last 15 days and the remaining 477 (7.2%) FSWs did not engage in sex work in those 15 days. Detailed data were documented on the last three clients for 5472 (82.4%) FSWs, on last two for 408 (6.1%), and on one client only for 288 (4.3%). In total, data were available on 17529 clients of 6171 FSWs who had at least one client in the last 15 days. Client characteristics as reported by the FSWs are summarised in Table 2.

**Table 2:** Distribution of client characteristics as reported by the FSWs.

**Variable**	**Variable categories**	**Number (%) (N = 17529)**
Type of client*	New	12047 (68.7%)
	Regular	5459 (31.1%)
	Do not remember	23 (0.2%)

Age of client	Young	8639 (49.3%)
	Middle-aged	8468 (48.3%)
	Old	272 (1.6%)
	Cannot say	150 (0.8%)

Marital status of client	Single	4785 (27.3%)
	Married	9358 (53.4%)
	Do not know	3386 (19.3%)

Economic status of client	Poor	3265 (18.6%)
	Average	9649 (55.0%)
	Wealthy	2535 (14.5%)
	Cannot say	2080 (11.9%)

Truck driving as profession of the client	Yes	1461 (8.3%)
	No	13217 (75.4%)
	Cannot say	2851 (16.3%)

Client highly intoxicated with alcohol/ drugs	Yes	1015 (5.8%)
	No	16447 (93.8%)
	Do not remember	67 (0.4%)

*New client was the one who visited the FSW for the first time, and regular client was the one who visited the FSW repeatedly.

### Use of condom between FSWs and their clients

Of the 6171 FSWs who had at least one client in the last 15 days, 6165 (99.9%) had had penetrative vaginal/anal sex with at least one client. This penetrative sex was predominantly vaginal with only 12 (0.2%) and 49 (0.8%) reporting anal and oral sex with clients – the anal/oral sex was in addition to vaginal sex. 2907 (47.2%; 95% CI 41.2–53.2%; design effect 23.0) FSWs had either not used condom at all or not used with all the clients with whom penetrative sex was done (for a maximum of the last 3 clients on whom data were available). The proportion of no or inconsistent use of condom with clients was 53.7%, 30.2% and 13.3% for the street-, home- and brothel-based FSWs, respectively.

With multiple logistic regression analysis (Table 3), the highest odds ratio predicting for no or inconsistent use of condom for penetrative vaginal/anal sex were for FSWs who did not know that HIV could be prevented, followed by those who did not have access to free condoms in the last 30 days, who did not participate in FSW support group, street- and home-based FSWs, and those who had low social support score. The other variables significantly associated with condom non-use are shown in Table 3. We also assessed the interactions between some variables in another logistic regression model. Knowledge that HIV can be prevented interacted significantly with access to free condoms in the last 30 days, education level of FSW, and rural-urban area where the FSW was sampled from (p < 0.001); and there was also significant interaction between participation in FSW support group and social support score (p < 0.001). The level of knowledge that HIV can be prevented varied significantly among the FSWs among the 40 geographic sites ranging from 14.1% to 95.2%, and there was a direct linear correlation between this knowledge and consistent use of condom (p < 0.001) (Figure 1).

**Table 3:** Association of select variables with no or inconsistent use of condom for penetrative vaginal/anal sex by FSWs with their clients in multiple logistic regression.

Variable*	**Variable categories†**	**Total who had penetrative sex with at least one client (6,128)‡**	**Number who reported no or inconsistent use of condom (%)**	**Odds of having no or inconsistent use of condom (95% CI)**
Knowledge that HIV can be prevented	Yes	3321	840 (25.3%)	1.00
	No	2807	2067 (73.6%)	5.01 (4.38–5.73)

Access to free condoms in the last 30 days	Yes	2468	501 (20.3%)	1.00
	No	3660	2406 (65.7%)	3.45 (2.99–3.98)

Participation in FSW support group	Yes	566	77 (13.6%)	1.00
	No	5561	2829 (50.9%)	2.02 (1.50–2.70)

Type of sex worker	Street-based	4599	2468 (53.7%)	3.36 (1.87–6.04)
	Home-based	1394	421 (30.2%)	2.66 (1.46–4.86)
	Brothel-based	135	18 (13.3%)	1.00

Social support score§	1.00 – 2.50	1306	836 (64%)	2.60 (2.17–3.12)
	2.51 – 3.50	2518	1387 (55.1%)	2.27 (1.95–2.64)
	>3.50	2304	684 (29.7%)	1.00

Income in the last 7 days (Rupees)	>501	1903	515 (27.1%)	1.00
	251 – 500	1929	946 (49%)	1.31 (1.09–1.57)
	250 or less	2295	1445 (63%)	1.66 (1.35–2.04)

Age group (years)	16 – 24	2292	875 (38.2%)	1.00
	25 – 34	3034	1533 (50.5%)	1.29 (1.11–1.51)
	35 or more	802	499 (62.2%)	1.69 (1.33–2.14)

Family aware of sex work	Yes	2064	655 (31.7%)	1.00
	No	4061	2250 (55.4%)	1.32 (1.13–1.53)

Rural-urban area where the FSW was sampled from	Rural	1345	544 (40.4%)	1.45 (1.19–1.77)
	Urban small	680	266 (39.1%)	1.07 (0.84–1.35)
	Urban medium	2621	1460 (50.2%)	1.73 (1.47–2.04)
	Urban large	1482	637 (43%)	1.00

Number of clients in last 7 days	7 or less	3768	2062 (54.7%)	1.20 (0.88–1.63)
	8 – 14	1829	738 (40.3%)	1.25 (0.93–1.67)
	> = 15	531	107 (20.2%)	1.00

Marital status	Never married	1072	257 (24%)	1.00
	Currently married	2483	1327 (53.4%)	1.26 (1.01–1.59)
	Other#	2573	1323 (51.4%)	1.07 (0.85–1.35)

Education level of FSW	Illiterate	4552	2428 (53.3%)	1.32 (1.13–1.55)
	Literate	1576	479 (30.4%)	1.00

Duration of being in sex work (years)	0–5	4773	2336 (48.9%)	1.00
	>5	1355	571 (42.1%)	0.98 (0.82–1.17)

*Variables listed in descending order of effect on outcome variable.

†Some categories of variables combined based on initial iterations that showed similar values for outcome variable in order to increase the power of the analysis.

‡Data on condom use was available for 6128 (99.4%) FSWs; the total of sub-categories may not always be 6128 due to a few missing values.

§The social support score for each respondent was averaged for responses to 7 questions used for this score, which documented whether the respondent could count on someone for money, going to doctor, talking about problems, food or place to stay, abuse by anyone, abuse by client, and client's refusal to use condom; this score ranged from 1 to 5, with 1 indicating least social support and 5 indicating maximum social support; the three suggested categories indicate low, medium and high social support, respectively.

¶Urban small were towns with population < 50,000; urban medium were towns/cities with population 50,001 – 200,000; urban large were cities with population more than 200,000; this classification was done based on Census of India data for each sub-site.

#Other includes separated, divorced and widowed.

**Figure 1:**
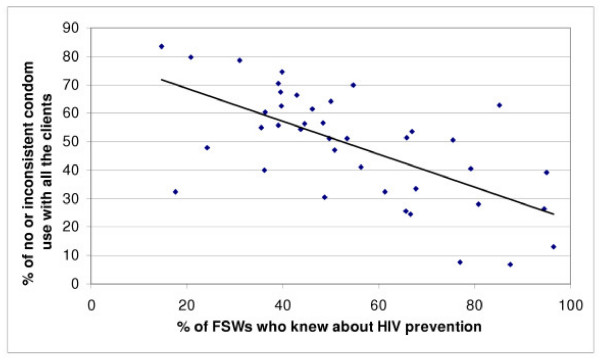
Relation between FSWs having knowledge that HIV could be prevented and inconsistent or no use of condom with clients for penetrative vaginal/anal sex in the 40 geographic sites.

#### Dynamics of condom use

A total of 17517 (99.9%) clients had penetrative vaginal/anal sex with FSWs. Of these clients, 10860 (61.9%) used condom, 6418 (36.6%) did not use, and information on condom use was not available for 239 (2.5%) clients. Among the 10860 clients who had used condom – FSWs had suggested using condom to 6816 (62.8%) clients, and of these 2697 (39.6%) clients had to be convinced to use condom; 2070 (19.1%) clients themselves had suggested using condom; and both the FSWs and clients had suggested using condom with 1971 (18.1%) clients. Condom was supplied by FSWs for 7531 (69.3%) clients. Among the 6418 clients who did not use condom for penetrative sex, FSWs had condoms available with them at the time of sex for 830 (12.9%) of these clients.

Considering only the last client for each FSW with whom she had penetrative vaginal/anal sex, the clients who did not use condom were more likely to be middle-aged or old (p < 0.001), married (p < 0.001), and with lower economic status (p < 0.001) as reported by the FSWs.

### Use of condom between FSWs and their regular sex partners

A total of 3642 (54.8%) FSWs reported having a regular sex partner (*husband, lover, boyfriend*) of whom 2582 (70.9%) had had penetrative sex with him within the last 7 days from the day of interview. 2428 (94%; 95% CI 92.1–95.9%; design effect 4.3) FSWs had not used condom for the last penetrative sex with their regular sex partners. The major reason cited for not using condom was *do not use because he is my regular partner *(1802, 73.9%) followed by *not aware of condom *(364, 14.9%), *he does not like it *(178, 7.3%), *want to have children *(118, 4.8%), and others (not mutually exclusive).

Of the 2464 FSWs who had had penetrative vaginal/anal sex with at least one client in the last 15 days and also had penetrative vaginal/anal sex with their regular sex partner in the last 7 days, 1032 (41.8%) FSWs had neither used condom consistently with clients nor had used with regular sex partner and 1102 (44.7%) had used condom consistently with clients but not used with regular sex partner. The former group tended to be street-based FSW (p < 0.001), having no knowledge about HIV prevention (p < 0.001), more than 24 years of age (p = 0.003), ever married (p < 0.001), and illiterate (p < 0.001) as compared with the FSWs who had used condom consistently with their clients but had not used with their regular sex partner.

## Discussion

The national HIV prevalence in India is still estimated to be low but there is a serious HIV epidemic in six states where the majority of the infections are acquired sexually [1,2]. We have reported data on condom non-use for a large sample of FSWs from the state of Andhra Pradesh, where HIV prevalence among FSWs was estimated to be 16%, ranging from 8–41% in seven surveillance sites in 2004 [21]. These data on the different types of FSWs from various urban and rural areas of the state using a standard methodology would allow relatively broader understanding of issues that are relevant for HIV prevention programmes, and for promoting use of condom in particular.

The FSWs who participated in this study may not be representative of all FSWs as they were recruited through FSW facilitators suggesting a bias towards those who are better connected with their peers, and hence the results should be interpreted within this limitation. It is also possible that some respondents would have over-reported use of condom, therefore the actual use of condom may be lower than that reported.

### Non-use of condom

Nearly half of the FSWs had not used condom consistently with all the clients in this study. In another assessment in this state in year 2000, only 25.9% and 8.5% FSWs had reported use of condom with all clients in the preceding one-month [7]. From the perspective of HIV prevention programmes, we found substantial differences in use of condom between the different types of FSWs, with the street-based FSWs nearly 3.5 times less likely to use condoms with clients as compared with the brothel-based FSWs. The street-based FSWs are also the highest in proportion among the different types of FSWs in India, including Andhra Pradesh [22].

Knowledge that HIV can be prevented was a strong predictor of consistent use of condom for penetrative sex between FSWs and their clients. Another variable strongly associated with consistent condom use was access to free condoms. These findings reinforces that knowledge about HIV and access to free condoms are vital for promoting increased use of condoms in FSWs in India. In addition, the FSW demographic characteristics that predict inconsistent use of condom with their clients were – age more than 24 years, currently married, illiterate, lower income, poor social support, family unaware of sex work, and no participation in FSW support groups. These characteristics can be used to define target groups for HIV intervention programmes. Some client characteristics associated with non-use of condom were also identified which can be used to promote condom use by these vulnerable clients.

Condom use for penetrative sex with the regular sex partner was negligible, and 41.8% FSWs had neither used condom consistently with clients and nor had used with their regular sex partner in the last sexual act. It may be difficult to promote use of condom between FSWs and their regular sex partners as married and cohabitating couples, in general, use condom less frequently because of various reasons [23]. However, as is highlighted by these data a significant proportion of FSWs have unprotected sex with clients and their regular sex partners, and it is possible that the regular sex partners of FSWs are not necessarily monogamous. Therefore, HIV transmission from regular sex partners may increasingly contribute to the overall spread of HIV as the use of condom increases with the clients. These data have also highlighted the dynamics of condom use ranging from FSW convincing the client to use condom, the client convincing FSW to use condom, to condom being available with FSW but not used at the time of sex with client. Although the overall use of condom was low, when it was used it was primarily at the suggestion of FSW, though a significant proportion of clients also asked for the condom to be used. Further research is necessary to better understand whether the demand from client for use of condom or the ability of FSW to convince the client to use condom is more effective in promoting condom use.

### HIV prevention

#### Context and environment

Effective HIV prevention requires strategies and policies that help reduce vulnerability of FSWs to HIV infection by creating a social, legal and economic environment in which prevention is possible. In India, as elsewhere, creating an enabling environment for behaviour change among the individuals who are at a higher risk of acquiring or transmitting HIV is an integral part of the HIV interventions [13]. We discuss the findings of this study within this context for HIV prevention in sex workers in India.

It is estimated that about 1.1% of the adult women in India could be engaged in sex work [24], most of whom are estimated to be non-brothel based [22]. The non-brothel based FSWs, especially street-based, were at a higher risk of HIV infection as compared with brothel-based FSWs in this study. Because the Indian society discriminates against FSWs as immoral women, not many of them acknowledge that they are sex workers. Only one-third of FSWs in this study reported that their families were aware of their sex work. FSWs with lower social support score were relatively less likely to use condom consistently. These women, for the most part, remain inaccessible to HIV prevention programmes, thereby undermining the efforts of HIV prevention. Acknowledgment of being a sex worker is more of an issue with the non-brothel based FSWs as compared with the brothel-based FSWs because being in a brothel can be interpreted as an acknowledgment that she is a sex worker.

This also makes it difficult to organize non-brothel based FSWs as a group that could be empowered to protect themselves and participate in the HIV prevention efforts. A model from India has been reported to be successful in empowering FSWs in Sonagachi, Kolkatta [11,25]. However, there are also examples of not so successful peer-based HIV interventions in brothel-based FSWs in Mumbai, India who were not interested in conducting education sessions for their peers after being trained, and their madams did not allow them to leave the brothels to conduct education programmes [26]. Only 9% of FSWs in our study reported participation in FSW support group, and these women reported higher use of condom with clients. Even though the number of brothel-based FSWs in this study was small, these data highlight that the risk behaviour for HIV was lower in the brothel-based FSWs as compared with the others, thereby, suggesting that sex workers working together as a group can promote condom use with clients.

Accessibility to and empowerment of non-brothel based FSWs is also very closely linked to the legal environment related to sex work in the country. As recently as June 2004, a participatory intervention programme for HIV prevention among FSWs in Goa, India was put to an abrupt end because the Government of Goa demolished the red-light area of FSWs in its effort to eradicate prostitution and rehabilitate FSWs [27]. The women displaced from this area reported rape, increased violence, reduced ability to negotiate condom use, and multiple partners following this act [27]. The legal context of sex work in India is quite complex, and FSWs are held by police under the *Immoral Traffic Prevention Act *that deals with human trafficking [28]. Prostitution by itself is not a crime under this Act unless it amounts to nuisance but prostitution-related activities such as running a brothel, making a living on prostitution earnings of another person, or procuring a person for prostitution are a crime. In reality, this Act is more often used to *book *sex workers and not pimps or clients and is also a source of corruption for the police [29,30], and impedes the provision of HIV prevention for FSWs [27].

In terms of economic vulnerability, the mean income per day for FSWs in this study ranged from Rs. 82, Rs. 135, and Rs. 174 for street-, home- and brothel-based FSWs, respectively. The FSWs with income of Rs. 500 or less in a week were more likely not to use condom consistently with all clients. Therefore, within the context of negligible social empowerment, lack of organized FSW groups, less number of paying clients to earn money, and lower income, it is not always feasible for FSWs to demand the use of condom with the clients.

#### Approach to prevention

With increased annual budget for the National AIDS Control Programme, expansion of antenatal screening, increased provision of anti-retroviral treatment, and constitution of a National Parliamentarian Forum to generate political support for HIV programmes, the HIV epidemic is one of the top national public health priorities in India [31]. Significant HIV prevention interventions for FSWs are currently on-going in India [13], and would continue to be expanded to increase the coverage of HIV prevention programmes. However, many of the examples of HIV interventions in sex workers available from India are for brothel-based sex workers, and not many for the non-brothel based sex workers who are the majority in the country. Data from this study have indicated significant differences between the brothel- and non-brothel-based FSWs in terms of risk of HIV infection, and therefore, the HIV prevention efforts require strategies to access non-brothel-based sex workers in order to narrow down these differences. The context and environment is also different for the brothel- and non-brothel-based FSWs. The efforts to expand coverage of HIV prevention activities amongst sex workers will depend on achieving and sustaining an environment that enables HIV prevention, which in turn is dependent on the sensitivity of these efforts to the varied contexts of these women. Some examples of such attempts are available globally, including from India [8,25]. There are also lessons to be learnt if the prevention efforts do not involve sex workers as primary stakeholders in these programmes [32,33] or when legal environment disrupts the prevention efforts [27].

## Conclusion

It seems necessary that the HIV prevention programmes with female sex workers in India expand beyond generic programmes to be tailored effectively to reach the different types of female sex workers in their local context, especially non-brothel based female sex workers. More comprehensive prevention efforts are needed that include changing the social and legal context of sex work, which would create an environment for sustained reduction of HIV risk in female sex workers.

## Competing interests

The author(s) declare that they have no competing interests.

## Authors' contributions

RD contributed to the study design, data collection, data analysis and interpretation, and drafted the manuscript. LD contributed to the study design, data collection, and data analysis and interpretation. JPG contributed to the study design, data interpretation, and coordination. GAK contributed to the data management, analysis and interpretation. SM, FS and SMB contributed to the study design, data interpretation and coordination. All named authors read, commented on and approved the final version of the manuscript. The ASCI FPP Study Team contributed to the planning of the study logistics, data collection and interpretation, and the members of this Team other than the named authors include (in alphabetical order): G Md Mushtaq Ahmed, Md Akbar, Md Abdul Ameer, Ch Arjun, N Arjun, M Sai Baba, C Satish Babu, J Kishore Babu, I Balasubrahmanyam, V S Udaya Bhaskar, T Gangadhar, P Gopal, Lavanya Gotety, Shaik Omar Hussain, V Indira, S Krishna, P Kiran Kumar, Ch Sri Jaya Lakshmi, T Uma Maheshwar, P Chandra Mouli, S Radhakrishnan, K Raghu, S P Ramgopal, A Srinivas Rao, A Srinivasa Rao, K Hanumantha Rao, N Ananda Rao, P Venkateswara Rao, Parsa V R Rao, D Ravinder, A Srinivas Reddy, G Brahmananda Reddy, S Krishna Reddy, G Uma Sankar, A Satyam, Y S Sivan, P V Sridhar.

## Pre-publication history

The pre-publication history for this paper can be accessed here:


